# A Ratiometric Fluorescent Sensor for Penicillin G Based on Color-Tunable Gold–Silver Nanoclusters

**DOI:** 10.1021/acsomega.3c09010

**Published:** 2024-02-20

**Authors:** Yu-Hung Yeh, Yu-Shen Lin, Tai-Chia Chiu, Cho-Chun Hu

**Affiliations:** Department of Applied Science, National Taitung University, No. 369, Sec. 2, University Road, Taitung City, Taitung County 95092, Taiwan (R.O.C.)

## Abstract

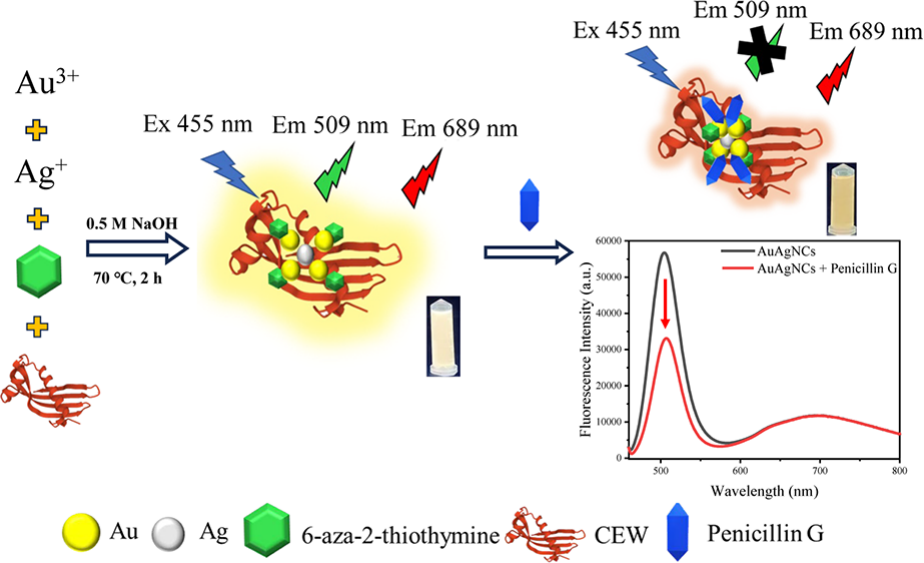

Excessive administration of penicillin G and improper disposal of its residues pose a serious risk to human health; therefore, the development of convenient methods for monitoring penicillin G levels in products is essential. Herein, novel gold–silver nanoclusters (AuAgNCs) were synthesized using chicken egg white and 6-aza-2-thiothymine as dual ligands with strong yellow fluorescence at 509 and 689 nm for the highly selective detection of penicillin G. The AuAgNCs were characterized using transmission electron microscopy, X-ray photoelectron spectroscopy, ultraviolet–visible absorption spectrophotometry, and fluorescence spectrophotometry. Under optimum conditions, the fluorescence intensity decreased linearly with the concentration of penicillin G from 0.2 to 6 μM, with a low detection limit of 18 nM. Real sample analyses indicated that a sensor developed using the AuAgNCs could detect penicillin G in urine and water samples within 10 min, with the recoveries ranging from 99.7 to 104.0%. The particle size of the AuAgNCs increased from 1.80 to 9.06 nm in the presence of penicillin G. We believe the aggregation-induced quenching of the fluorescence of the AuAgNCs was the main mechanism for the detection of penicillin G. These results demonstrate the ability of our sensor for monitoring penicillin G levels in environmental and clinic samples.

## 1 Introduction

Penicillin G, a β-lactam compound, is an antibiotic commonly and wildly used to cure bacterial infections in aquaculture, cattle, poultry, and humans.^1^ Unfortunately, the excessive administration of penicillin G to treat animals and the improper disposal of the corresponding residues lead to high residue levels in the environment and even livestock products which have the potential to damage human health.^2−6^ Of all the antibiotics, penicillin G may have the greatest potential for producing allergic responses to the consumer of food animal products.^7^ Moreover, prolonged or continuous administration of antibiotics to animals can cause antibiotic resistance.^8^ Therefore, it is a very important issue to monitor penicillin G in the environment and clinic samples.^9^ Various methods, including high-performance liquid chromatography and liquid chromatography–mass spectrometry, have been developed for the detection of penicillin G^10−14^ as well as immunoassay methods.^15,16^ However, these methods are very complicated, expensive, and time-consuming.^17^ Meanwhile, ratiometric fluorescence sensing, which is based on measuring the relative fluorescence intensities ratio at two emission wavelengths, is being increasingly applied for the analysis of the natural environment and living systems.^18−20^ In particular, precious-metal fluorescent nanoclusters have received extensive research attention as ratiometric fluorescence sensors in the past few years due to their excellent optical properties and biocompatibility.^21−23^ In contrast to single-signal detection, this method has built-in corrections for fluctuations in instrument operation, interference from the sample matrix, variations in the microenvironment around the probe, and changes in the concentration of the probe.^24^ Recently, biomass-derived materials have been used as protective and reducing agents for the design of nanomaterials with special optical properties, more biocompatibility, and less environmental influence.^25−28^ For instance, 6-aza-2-thiothymine (ATT) containing both amino and imino groups endows the corresponding nanoclusters with good biocompatibility.^23^ In addition, chicken egg white (CEW) is used in post-synthesis surface modification to stabilize gold nanoclusters (AuNCs) within the protein molecules.^29,30^ Moreover, doping AuNCs with silver (Ag) has been demonstrated to improve the cluster stability and selectivity in detection applications.^31−33^

Here, we synthesized noble Au–Ag bimetallic nanoclusters (AuAgNCs) stabilized and reduced by using ATT and CEW with yellow photoluminescence. On the basis of the selective quenching of the photoluminescence of the AuAgNCs in the presence of penicillin G, we developed a ratiometric fluorescence sensor for examining penicillin G using the emission fluorescence intensities at 689 and 509 nm as a reference signal and a response signal, respectively. Additionally, the mechanism of detection of Penicillin G by AuAgNCs has also been explored in detail. From our study, this fluorescence probe has demonstrated the practical applications in the real samples and we believe there are much other potential uses that could be developed for this sensor.

## 2 Materials and Methods

### 2.1 Chemicals and Materials

HAuCl_4_·3H_2_O, ATT, H_3_PO_4_, NaH_2_PO_4_, Na_2_HPO_4_, Na_3_PO_4_, chloramphenicol, Oxytetracycline, doxycycline, Norfloxacin, cefadroxil, Lincomycin, chlortetracycline, ciprofloxacin, Tylosin, monensin sodium, Ofloxacin, tetracycline, penicillin G, sodium hydroxide (NaOH, 99%), and other salts were bought from Sigma-Aldrich. All aqueous solutions are diluted by ultrapure water (ddH_2_O, ≥18.2 MΩ cm^–1^).

### 2.2 Apparatus

The RF-6000 fluorescence spectrophotometer (Shimadzu, Kyoto, Japan) was used to obtain the fluorescence spectra. UV–vis spectra were recorded from a U-2900 spectra-photometer (Hitachi, Tokyo, Japan). FT-IR spectra of the nanocluster and the origin ligands were performed on the Nicolet iS5 using the KBr-pellet fabrication. The X-ray photoelectron spectroscopy (XPS) spectra were carried out on a K-alpha XPS (Thermo Fisher Scientific, Waltham, MA, USA). Zetasizer Nano ZS9 (Malvern, Worcestershire, UK) was used to measure the zeta potentials and dynamic light scattering of our materials.

### 2.3 Synthesis of AuAgNCs

The egg white was separated from the whole egg and then freeze-dried to obtain a white powder as described in the literature.^34^ The CEW powder was used without any further purification. 0.1 M, 1 mL of NaOH solution mixed with ATT solution was vigorously stirred in the dark for 5 min. Meanwhile, various ratios of HAuCl_4_ and AgNO_3_ were stirred with an aqueous solution containing various concentrations of CEW. After 1 min, this solution was poured into NaOH solution and vigorously stirred in the dark for 5 min. The ATT solution was then mixed with metal ion solution. The gold–silver solution was heated for 2 h at 70 °C. Finally, the product AuAgNCs were kept in storage at 4 °C.

### 2.4 Fluorescence Detection of Penicillin G

Chloramphenicol, oxytetracycline, doxycycline, norfloxacin, cefadroxil, lincomycin, chlortetracycline, ciprofloxacin, tylosin, monensin sodium, ofloxacin, tetracycline, and penicillin G were evaluated by AuAgNCs and AuNCs to check selectivity of antibiotics. 200 μL of AuNCs or AuAgNCs was mixed with different antibiotics (200 μL, 10 μM) followed by adding 1600 μL of ddH_2_O. Fluorescence spectra were obtained by excited at 455 nm wavelength. The interaction between our sensor and antibiotics was assessed by the fluctuation in the fluorescence intensity ratio (*F*/*F*_0_). *F*_0_ and *F* were the fluorescence intensity of AuAgNCs at 509 nm (for AuNCs at 521 nm) without and with the antibiotics.

### 2.5 Determination of Penicillin G in the Real Samples

The practical applications of our nanosensor were confirmed by monitoring Penicillin G in the tap water and urine samples. The supernatant of water and urine samples was used after centrifugation (10,000 rpm, 15 min) and through a 0.22 μm nitrocellulose membrane to remove large suspended particles. The penicillin G solution was spiked into real samples, and the sensitivity of the system was subsequently confirmed.

## 3 Results and Discussion

### 3.1 Synthesis and Characterization of AuAgNCs

AuAgNCs were synthesized using different concentrations of CEW and ATT and various mole ratios of HAuCl_4_-to-AgNO_3_. The fluorescence intensities of the obtained samples are summarized in Table S1 and Figure S1. First, to explore the optimum concentration of CEW, AuNCs were synthesized using various concentrations of CEW at a fixed ATT concentration of 20 mM. The highest fluorescence intensity was obtained for a CEW concentration of 50 mg/mL. Various HAuCl_4_-to-AgNO_3_ ratios were also evaluated by examining the change in the fluorescence ratio *F*_509_/*F*_689_, finding that it was the closest to unity (*F*_509_/*F*_689_ = 2.09) when the HAuCl_4_-to-AgNO_3_ mole ratio was 8:2. Therefore, 8:2 was selected as the optimal mole ratio for the reaction system.^34^

**Figure 1:**
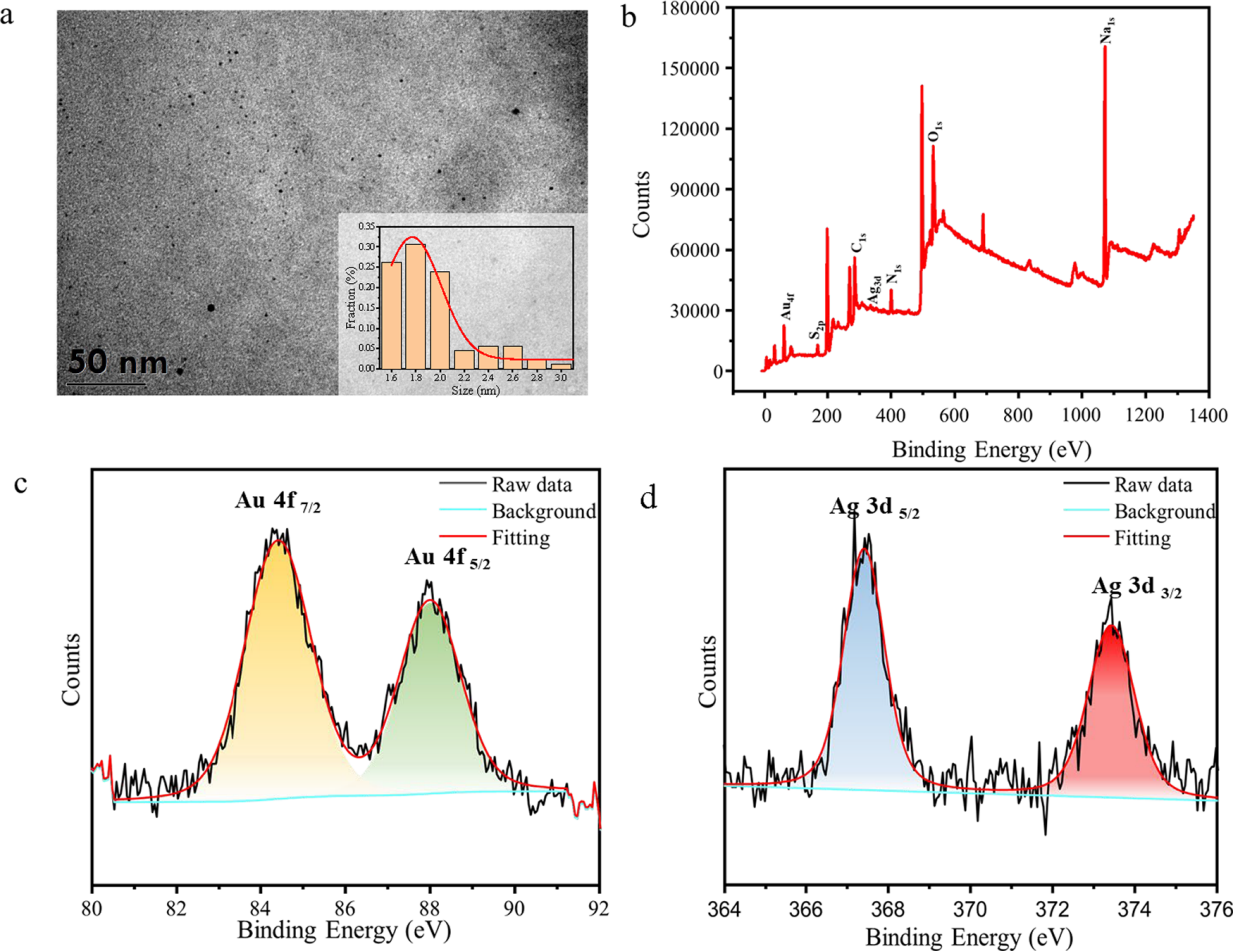
Characterization of AuAgNCs: (a) TEM images and size distribution, (b) survey XPS spectrum, and (c) Au 4f and (d) Ag 3d fine XPS spectra.

The transmission electron microscopy (TEM) image shown in Figure 1a revealed that the synthesized AuAgNCs were monodispersed and exhibited a spherical morphology with an average particle size of about 1.8 nm ± 0.18 nm. The element composition of the AuAgNCs was determined via XPS. As shown in Figure 1b, the XPS spectrum of the AuAgNCs displayed six characteristic peaks of Au 4f, Ag 3d, C 1s, N 1s, O 1s, and S 2p. The high-resolution Au 4f spectrum (Figure 1c) showed peaks at 88.0 and 84.5 eV, which can be assigned to Au 4f_5/2_ and Au 4f_7/2_, respectively.^35^ Meanwhile, two fitting peaks attributable to Ag 3d_5/2_ (Ag(I)) and Ag 3d_3/2_ (Ag(0)) were observed at 367.6 and 373.4 eV, respectively, in the Ag 3d XPS spectrum (Figure 1d).^36^ C 1s peaks showing the typical values of the C=O, C=N, and C–C groups of ATT appeared at 287.8, 286.2, and 284.3 eV, respectively (Figure S2a).^37^ Furthermore, the presence of O 1s peaks corresponding to H–O–H and O=C groups at 537.1 and 532.4 eV demonstrated that the as-prepared AuAgNCs were capped with ATT (Figure S2b). Meanwhile, the N 1s spectrum (Figure S2c) could be divided into three characteristic peaks located at 400.6, 399.7, and 399.1 eV, which were ascribed to Ag–N, N–H, and N=C bonds, respectively. The S 2p spectrum (Figure S2d) exhibited a peak at 162.5 eV attributable to Au–S covalent bonds. These observations demonstrate the presence of both ATT and CEW on the AuAgNCs surface.^38^

**Figure 2:**
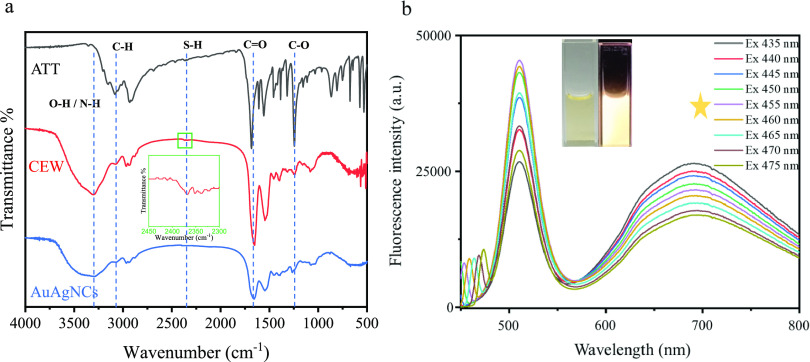
(a) FT-IR spectra of ATT (black), CEW (red), and the AuAgNCs (blue). (b) Fluorescence emission spectra of the AuAgNCs under different excitation wavelengths from 435 to 475 nm with an increment of 5 nm. The photos were AuAgNCs under the sun light and under the portable UV lamp.

The functional groups of the AuAgNCs were further investigated via Fourier transform infrared (FT-IR) spectroscopy. The FT-IR spectrum of ATT (black line in Figure 2a) showed peaks corresponding to the stretching vibration of C–O, C=O, and C–H at 1240, 1680, and 3090 cm^–1^, respectively.^38^ In the FT-IR spectrum of CEW (red line in Figure 2a), the peak at 3320 cm^–1^ can be attributed to the stretching vibration of the N–H and O–H groups, the peak at 3070 cm^–1^ corresponds to the stretching vibration of the C–H group, and the peak at 1660 cm^–1^ is ascribable to the stretching vibration of the C=O group.^39^ Moreover, a small band at 2370 cm^–1^ was observed in the FT-IR spectra of pure ATT and CEW. However, this band was not observed in the FT-IR spectrum of AuAgNCs, suggesting the formation of a direct covalent bond between the thiol group (−SH) and the AuAgNCs (Figure 2a).^40^

The optical properties of the AuAgNCs were investigated by recording their fluorescence spectra. Two fluorescence emission wavelengths were observed at 509 and 689 nm when the excitation wavelength was 455 nm (Figure 2b). The color of the AuAgNCs solution was light yellow under daylight irradiation, and a yellow fluorescence was emitted upon irradiation with 365 nm ultraviolet (UV) light (Figure 2b, **inset**). The absorption spectrum of the AuAgNCs was different from that of CEW and ATT (Figure S3).^41^ The UV–vis spectrum of ATT showed obvious absorption peaks at 215 and 269 nm, whereas no significant peaks were observed in this range in the spectrum of the AuAgNCs. Taken together, these characterization results confirmed the successful synthesis of the AuAgNCs.

### 3.2 Stability and Selectivity of the AuAgNCs

The effect of UV irradiation, ionic strength, pH, and storage time on the fluorescence stability of this sensor was explored. The fluorescence intensity of the AuAgNCs remained stable without significant changes upon storage at 4 °C for 1 month (Figure S4a). Stable fluorescence intensity was also observed after UV irradiation at 365 nm (UV lamp) or 455 nm (fluorescence spectrophotometer) for 60 min (Figure S4b,c).

Meanwhile, the fluorescence intensity of the AuAgNCs exhibited slight fluctuations as the NaCl concentration was increased to 200 mM (Figure S5a) and the fluorescence remained stable under the range of pH 8.0 to pH 10.0. In contrast, a decrease in the fluorescence intensity was observed under low pH conditions (pH 3.6–7.0), which might be attributed to the protonation of carboxyl groups inducing the aggregation of the AuAgNCs.^42^ In addition, the low fluorescence intensity might have been attributed to deprotonation under pH 11.0–12.0 (Figure S5b).^42−44^ The fluorescence intensity of the AuAgNCs was stable in water and methanol (Figure S5c) but was affected by highly polar solvents.^45^ The presence of different oxidizing or reducing agents caused slight fluctuations in the fluorescence intensity of the AuAgNCs (Figure S5d), indicating that they were not susceptible to oxidation or reduction. These results indicate that the AuAgNCs showed sufficient stability to be used as fluorescence labels for environmental and clinic samples.

**Figure 3:**
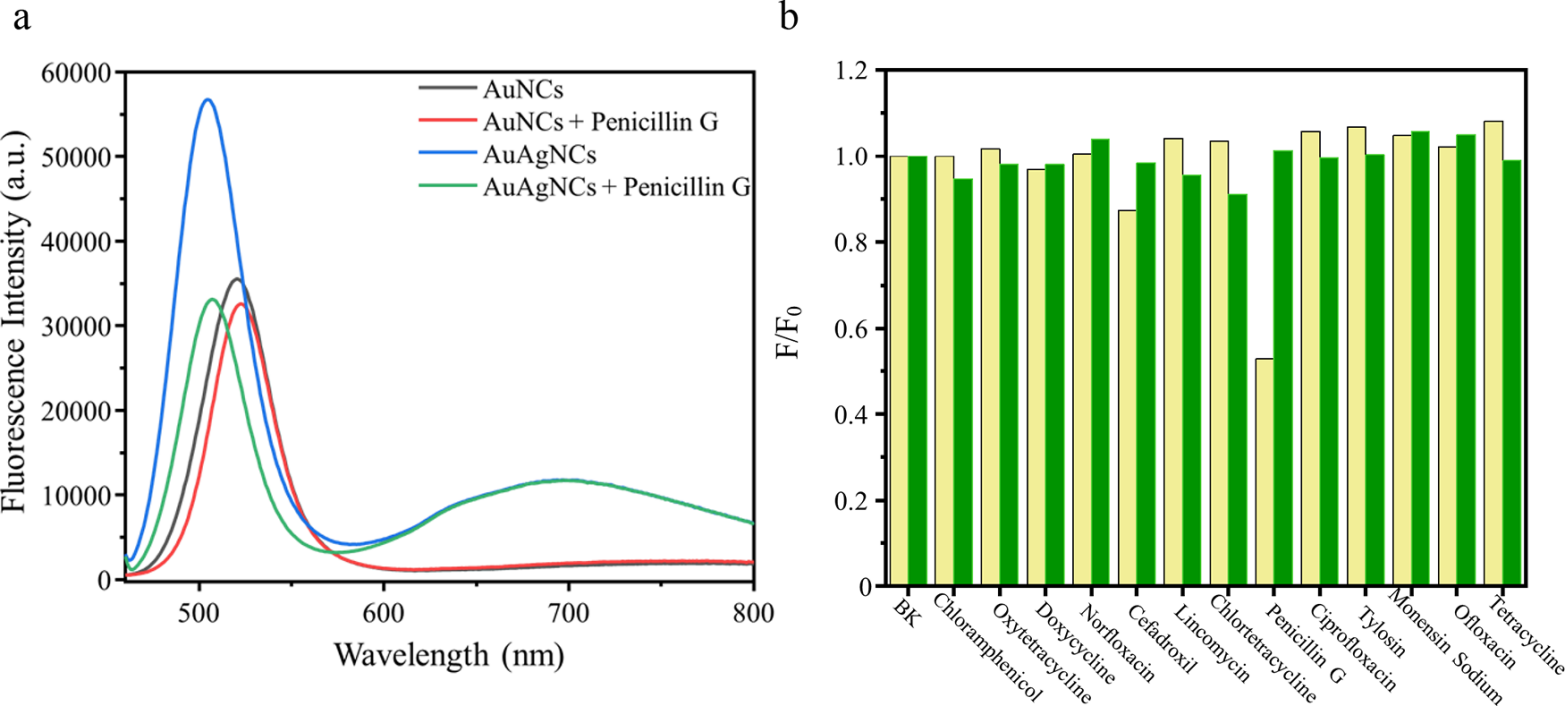
(a) Fluorescence emission spectra of the AuAgNCs and AuNCs before and after the addition of 10 μM penicillin G. (b) Selectivity of AuNCs (green) and AuAgNCs (yellow) toward multiple antibiotics (10 μM).

Figure 3a shows the effect of adding penicillin G on the fluorescence intensity of the AuNCs and AuAgNCs. Only the fluorescence intensity of the latter underwent a significant quenching; the fluorescence signal at 689 nm as a reference signal does not change significantly, most likely due to the “silver effect” on the Au interaction, causing a synergistic effect between Au and Ag in the AuAgNCs.^46,47^ In fact, some luminescent bimetallic AuAgNCs were previously fabricated by introducing Ag to change the luminescence of AuNCs.^46−48^Figure 3b demonstrates the selective quenching of the fluorescence intensity of the AuNCs and AuAgNCs in the presence of penicillin G compared with other antibiotics, i.e., chloramphenicol, oxytetracycline, doxycycline, norfloxacin, cefadroxil, lincomycin, chlortetracycline, ciprofloxacin, tylosin, monensin sodium, ofloxacin, and tetracycline, which exerted negligible effects on the fluorescence intensity of the AuAgNCs.

### 3.3 AuAgNCs as a Ratiometric Fluorescent Probe for Penicillin G Detection

**Figure 4:**
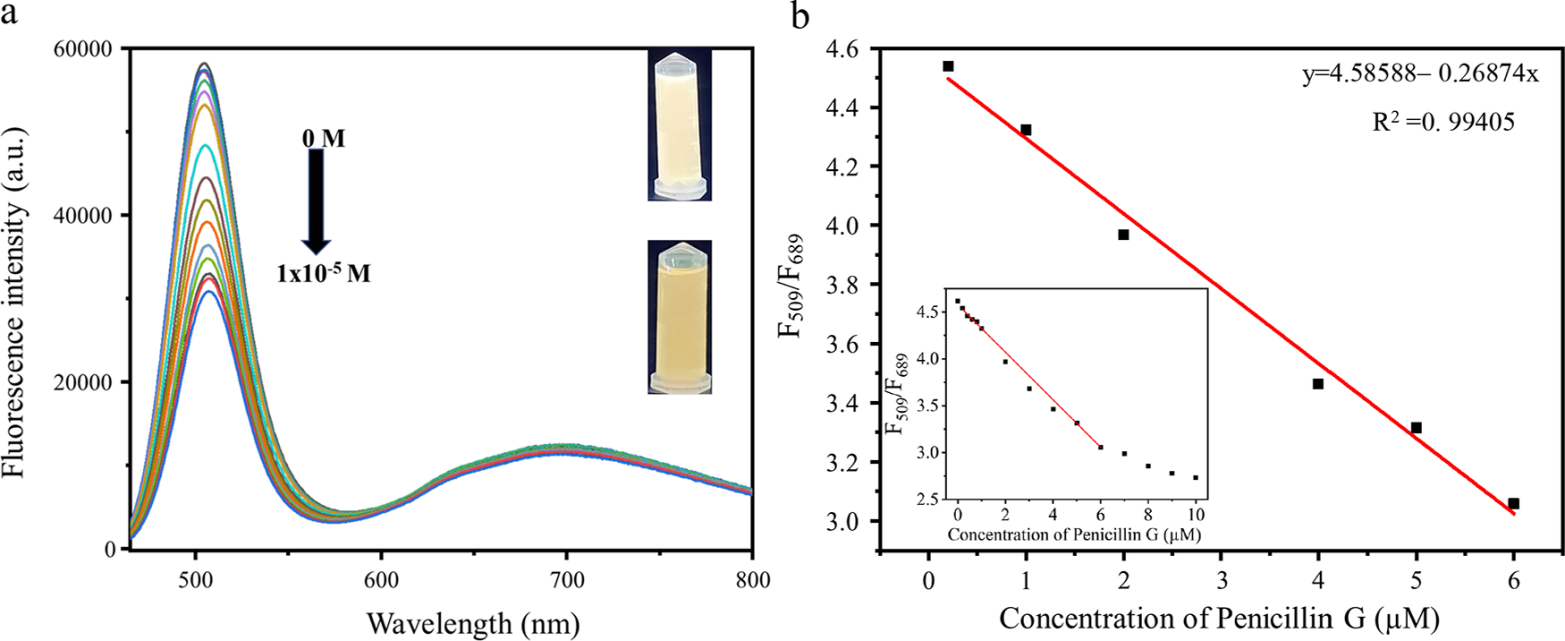
(a) Fluorescence spectra of the AuAgNCs at various penicillin G concentrations ranging from 0 to 10 μM. Inset: photographs of the corresponding AuAgNCs samples at penicillin G concentrations of 0 μM (top) and 10 μM (bottom) under portable UV lamp (b) Linear relationships between *F*_509_/*F*_689_ and the penicillin G concentration in the range of 0.2–6 μM (*n* = 3).

The incubation time and pH were optimized to obtain high sensitivity and a wide linear range of penicillin G detection. Figure S6a shows the results of the effect of the incubation time. The fluorescence was gradually quenched in the sensing system, reaching equilibrium at an incubation time of 10 min. As shown in Figure S6b, the quenching of the fluorescence intensity reached a maximum value at pH 8.0. Under these optimal sensing conditions of incubation time and pH, the fluorescence spectra of the AuAgNCs were recorded in the presence of various concentrations of penicillin G. As shown in Figure 4a, the fluorescence intensity of the AuAgNCs gradually decreased with increasing the penicillin G concentration. The presence of Ag on the AuAgNCs surface most likely played an important role in this assay. The −SH and carboxylic (−COOH) groups of penicillin G could complex with Ag, quenching the fluorescence at 509 nm. In contrast, no effect was observed on the fluorescence at 689 nm, suggesting that it stemmed from the core of the AuAgNCs and was more completely protected by the protein. Thus, the fluorescence signals at 689 and 509 nm could serve as a reference signal and a response signal for sensing penicillin G, respectively.

A good linear relationship was found between *F*_509_/*F*_689_ and the penicillin G concentration in the range of 0.2–6 μM with a correlation coefficient (*R*^2^) of 0.9941 (Figure 4b). The linear equation was as follows: *Y* = 4.58588 – 0.26874 [penicillin G (μM)]. The limit of detection (LOD) was calculated to be 18 nM based on 3σ/*s*, where σ represents the standard deviation of 10 blank measurements and *s* is the slope of the calibration curve.^49^

To explore the feasibility of this sensor, the as-developed fluorescent probes were used to detect penicillin G in tap water as well as urine. Table 1 summarizes the recoveries and relative standard deviations (RSDs) obtained at various penicillin G concentrations. Satisfactory recoveries between 99.7 and 104.0% with RSDs below 4.1% were obtained, demonstrating that the AuAgNCs-based sensor could reliably determine penicillin G in environmental and clinic samples.

**Table 1:** Detection of Penicillin G in Actual Samples (*n* = 3)

sample	spiked (μM)	found (μM)	recovery (%)	RSD (%)
tap water	0.50	0.50	100.0	0.41
	1.00	1.01	101.0	3.80
	3.00	2.99	99.7	0.58
urine samples	0.50	0.52	104.0	3.60
	1.00	1.02	102.0	3.30
	3.00	3.02	101.0	4.10

### 3.4 Mechanism of Penicillin G Sensing

**Figure 5:**
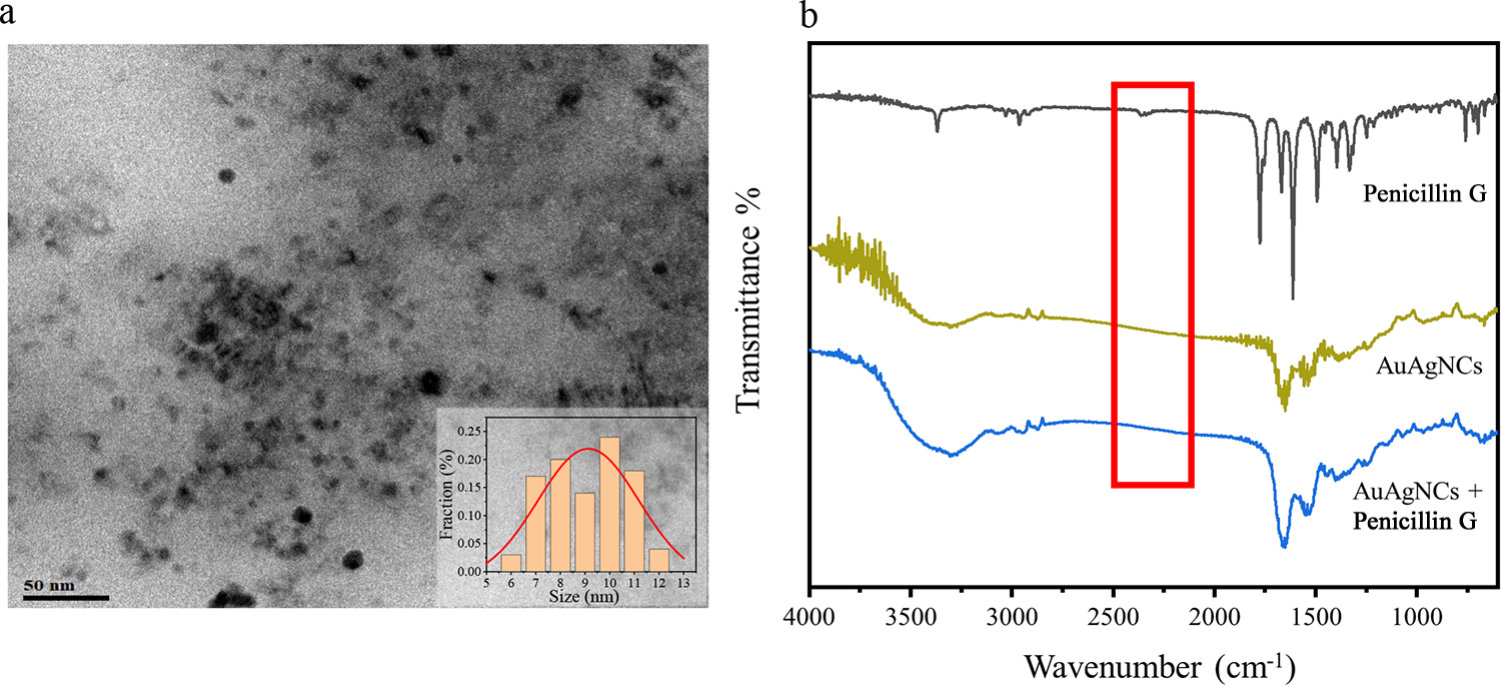
(a) TEM images and size distribution of the AuAgNCs in the presence of penicillin G. (b) FT-IR spectra of penicillin G (black), AuAgNCs (yellow), and AuAgNCs + penicillin G (blue).

Figure 5a shows the TEM images and size distribution of the AuAgNCs in the presence of penicillin G. The particle size of the AuAgNCs increased from the original 1.80 to 9.06 nm in the presence of penicillin G. Compared with a single fluorophore as a sensor, a ratiometric fluorescence probe with two emissions allows establishing a built-in self-calibration. The considerable quenching observed in the fluorescence emission spectra upon adding penicillin G might be assigned as aggregation-induced quenching^50^ resulting from the complexation of penicillin G and the AuAgNCs. The −SH and −COOH functional groups in penicillin G could coordinate with the AuAgNCs, inducing their aggregation.^51^

This hypothesis was confirmed via FT-IR spectroscopy. As shown in Figure 5b, the spectrum of penicillin G exhibited a peak attributable to −SH at around 2360 cm^–1^, whereas such a peak was not observed in the spectra of the AuAgNCs before and after the addition of penicillin G.^52^

The effect of penicillin G on the zeta potential of AuAgNCs was also investigated. Figure S7 shows the zeta potentials of the AuAgNCs without (−11.5 mV) and with (−19.0 mV) penicillin G. Considering that the zeta potential of penicillin G was −33.7 mV, it was estimated that penicillin G was bound to the AuAgNCs through strong coordination between Ag, Au, and S atoms.^53^ Compared with other reported fluorescent probes, our AuAgNCs sensor exhibited a shorter synthesis time, a lower LOD, and a shorter sensing time for penicillin G (Table S2).

## 4 Conclusions

In summary, AuAgNCs were synthesized using ATT and CEW as dual ligands. The AuAgNCs exhibited fluorescence emissions at 509 and 689 nm at an excitation wavelength of 455 nm, yellow fluorescence under a 365 nm UV lamp, and a pale-yellow color under daylight. The AuAgNCs remained stable under prolonged light exposure, high ionic strength, and different pH values. Using the AuAgNCs, a ratiometric sensing platform for the selective detection of penicillin G was constructed with the fluorescence emissions at 689 and 509 nm acting as a reference signal and a response signal, respectively. This ratiometric fluorescent sensor could effectively reduce the error due to interferences such as environmental changes, affording a more accurate detection result. The coordination of the–SH group in penicillin G with Au or Ag induced the aggregation of AuAgNCs molecules and, in turn, a decrease in the fluorescence intensity. Under the optimum conditions, the fluorescence intensity decreased linearly with the concentration of penicillin G in a range from 0.2 to 6 μM, and the LOD was 18 nM. The recovery rates of penicillin G in real tap water and urine samples were in the range of 99.7–104.0% with RSDs below 4.1%, revealing the high reliability and accuracy of the sensing system. Thus, the present work offers a facile, cost-effective, and highly stable sensing system for monitoring penicillin G residues in real samples with high sensitivity.

## Supporting Information Available

Optical characteristic of the AuAgNCs; stability of AuAgNCs; relative intensity (*F*/*F*_0_) of the mixture of AuAgNCs and penicillin G; zeta potentials of AuAgNCs and AuAgNCs with penicillin G; fluorescence intensity of AuNCs with different conditions of CEW; and comparison of the present study with other analytical methods (PDF)
