# Supplementary material for: A Ratiometric Fluorescent Sensor for Penicillin G Based on Color-Tunable Gold–Silver Nanoclusters
Source: ACS Omega. 2024 Feb 20;9(9):10621–7. doi: 10.1021/acsomega.3c09010 (PMC10918794; doi:10.1021/acsomega.3c09010)
Supplement: Supplementary file 1 — ao3c09010_si_001.pdf [file ao3c09010_si_001.pdf]

Supporting Information

A ratiometric fluorescent sensor for penicillin G based on color-tunable gold-silver nanoclusters

Yu-Hung Yeh, Yu-Shen Lin, Tai-Chia Chiu, Cho-Chun Hu*

Department of Applied Science, National Taitung University, No. 369, Sec. 2, University Road, Taitung City, Taitung County 95092, Taiwan (R.O.C.)

Corresponding Author

Department of Applied Science, National Taitung University, No. 369, Sec. 2, University Road, Taitung City, Taitung County 95092, Taiwan (R.O.C.)

Tel.: +886 (089)517991#6434. E-mail address: cchu@nttu.edu.tw

Table S1 The fluorescence intensity of AuNCs with different conditions of CEW.

CEW (mg/mL)	Excitation (nm)	Emission (nm)	Fluorescence Intensity (a.u.)
30	335	650	2968.6
40	335	650	8971.9
50 ★	335	650	11443.9
60	335	650	4673.6
70	335	650	3914.7

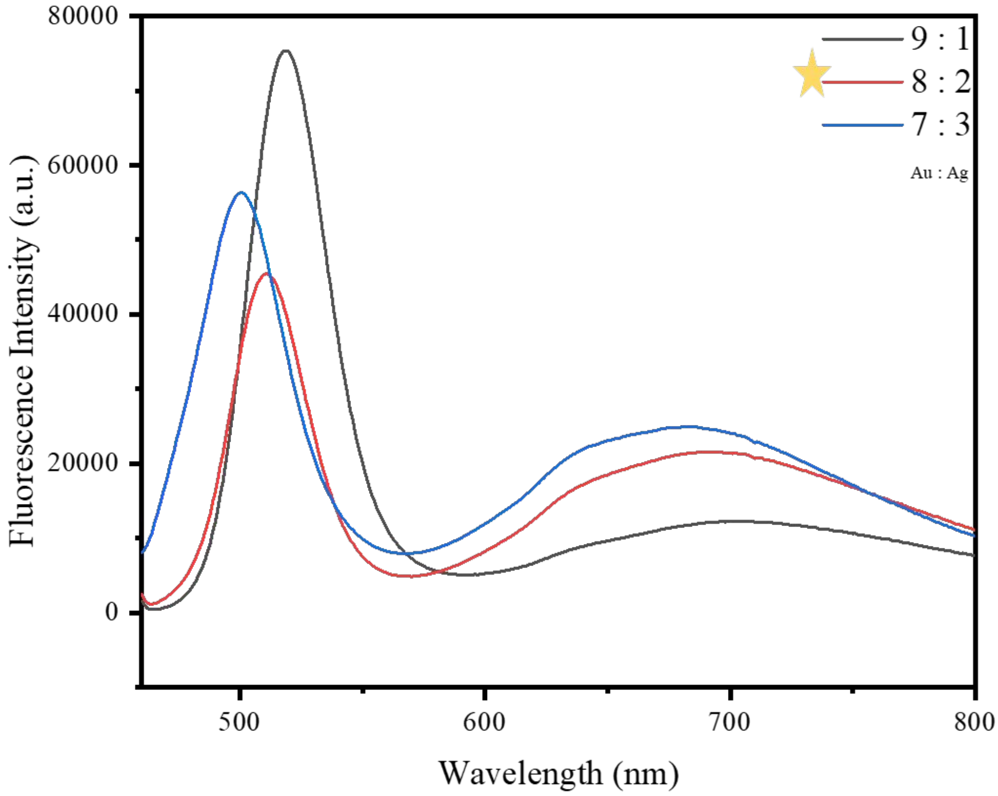

Figure S1 Fluorescence emission spectra of the AuAgNCs at various mole ratios of Au and Ag ions.

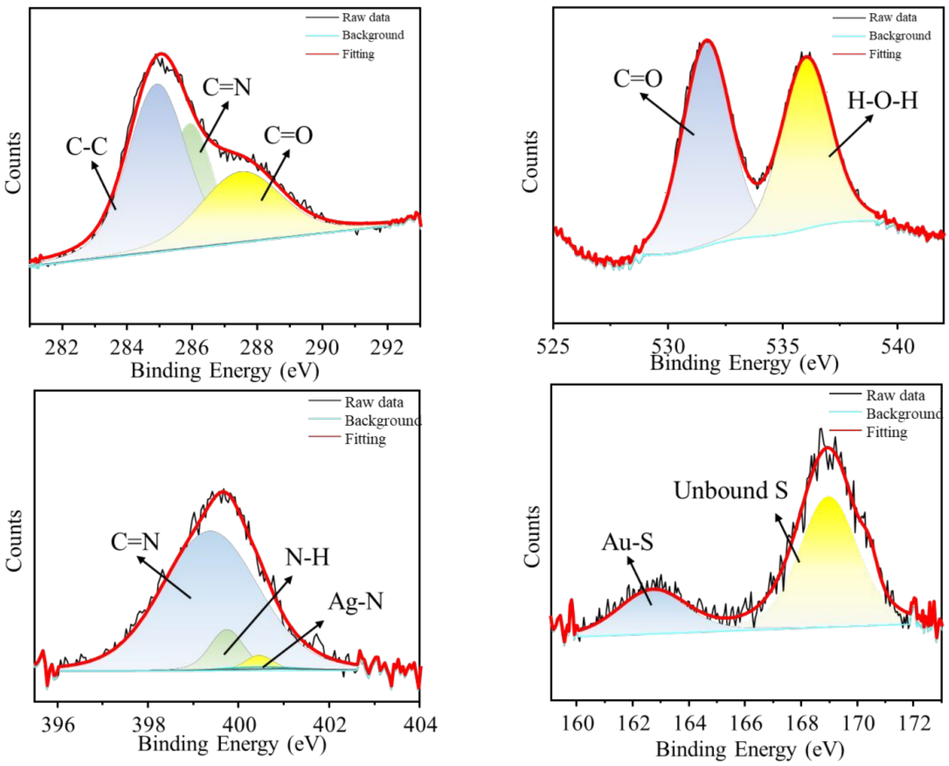

Figure S2 XPS spectra of AuAgNCs (a) C1s (b) O1s (c) N1s (d) S2p.

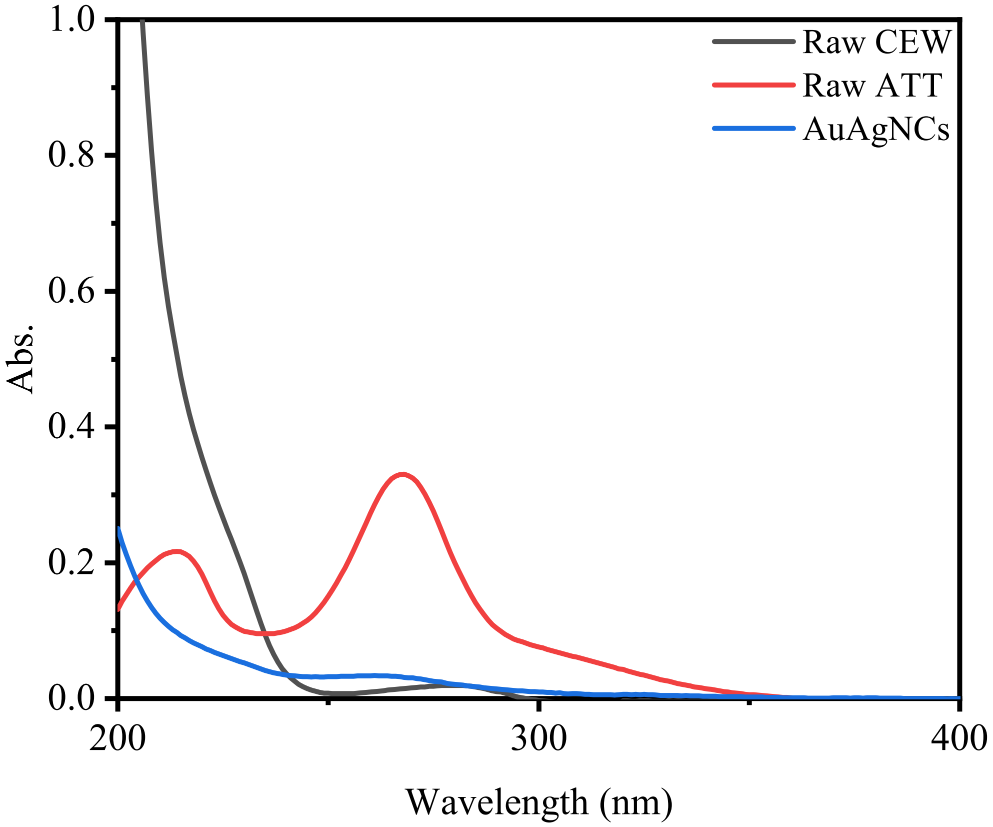

Figure S3 The UV-VIS absorption spectrum of CEW, ATT, and AuAgNCs.

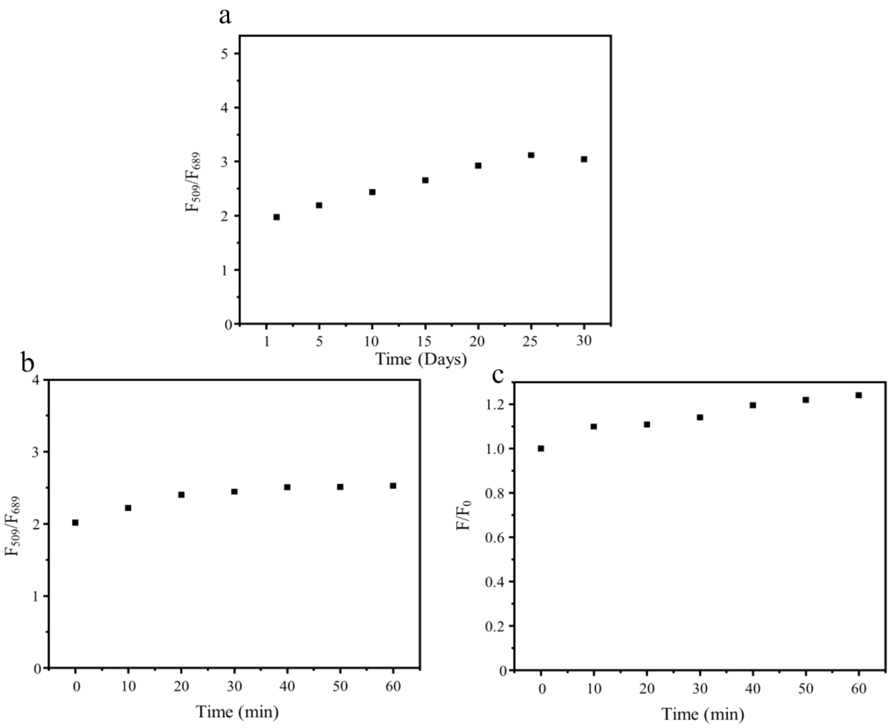

Figure S4 (a) The fluorescence stability AuAgNCs in 30 days. (b) Light stability of AuAgNCs by 365 UV lamp. (c) Light stability of AuAgNCs by fluorescence spectrophotometer.

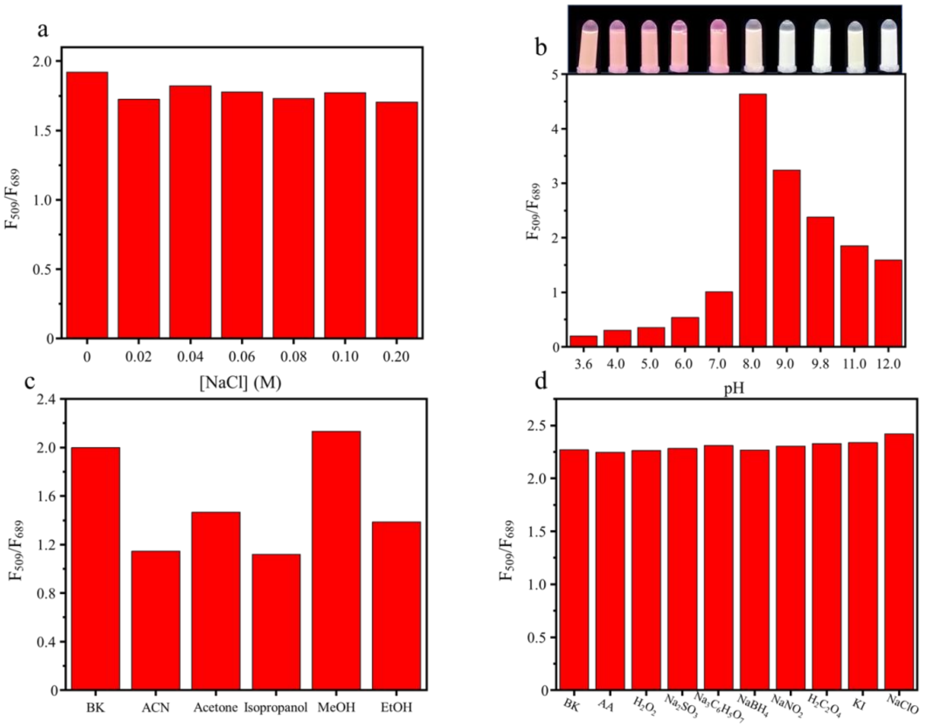

Figure S5 (a) Effect of the concentration of NaCl on the AuAgNCs. Fluorescence spectra of AuAgNCs in PBS buffers of different (b) pH values (c) solvent (d) oxidizing/reducing agents.

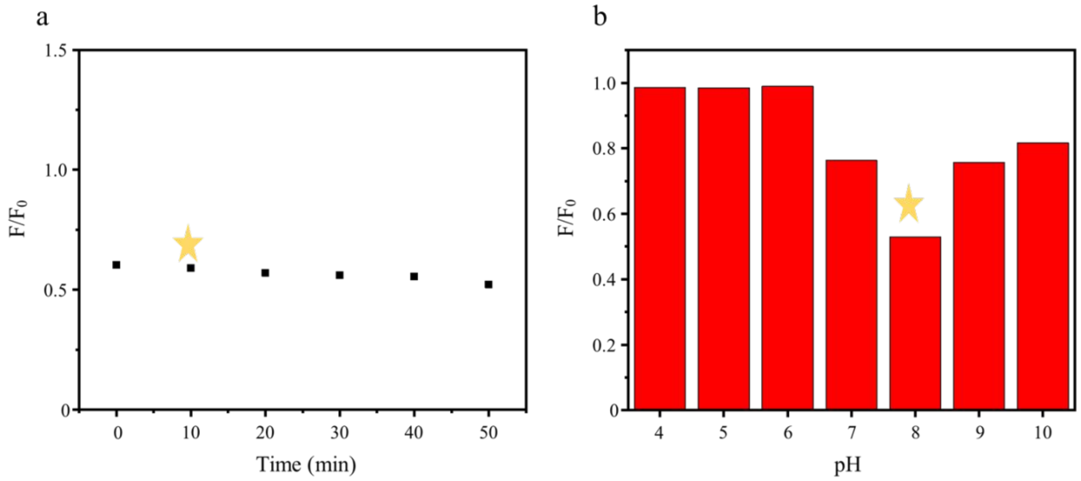

Figure S6 Effect of the (a) react time (b) react pH values on the relative intensity (F/F_0) of the mixture of AuAgNCs and Penicillin G (10 μM).

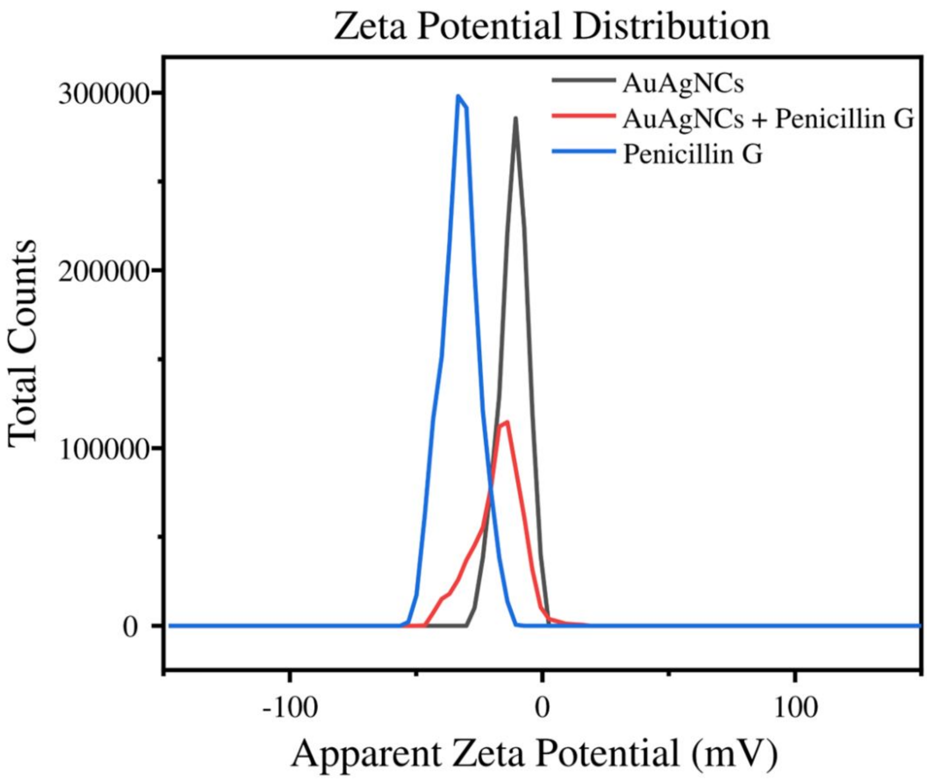

Figure S7 Zeta potentials of AuAgNCs (black)、Penicillin G (blue) and AuAgNCs + Penicillin G (red).

Table S2 Comparison of the present study with other analytical methods for the detection of Penicillin G.

Method	Sensor	Linear range (μM)	LOD (μM)	Ref.
Flow injection analysis–solid phase extractio(UV–vis) spectrometry	methylene blue grafted polyurethane foam sorbent	0.11–0.32	0.032	El-Shahat et al., (2010)[1]
(FTIR) spectrometry		8–85	1.3	Sadeghi et al., (2010)[2]
Chemiluminescence	potassium permanganate degradation	0.27–27	0.18	Cao et al., (2005)[3]
Chemiluminescence	H_2O_2 and N-cetyl-N,N,N-trimethylammonium bromide.	3.3–330	0.88	Sorouraddin et al., (2011)[4]
differential pulse voltammetry		0.5–40	0.25	Švorc et al., (2012)[5]
**Fluorescence**	**ATT-CEW-AuAgNCs**	**0.2-6**	**0.018**	**This work**

References

(1) El-Shahat, M. F.; Burham, N.; Azeem, S. M. A. Flow injection analysis–solid phase extraction (FIA–SPE) method for preconcentration and determination of trace amounts of penicillins using methylene blue grafted polyurethane foam. *Journal of Hazardous Materials* **2010**, *177* (1), 1054-1060. DOI: https://doi.org/10.1016/j.jhazmat.2010.01.027.

(2) Sadeghi, M.; Zeeb, M.; Kalaee, M. R. On-line Vapor-Phase Generation Followed by Fourier-Transform Infrared Spectrometry for the Quantitative Analysis of Water-Soluble Penicillin G in Pharmaceutical Formulations. *Analytical Sciences* **2010**, *26* (5), 575-580. DOI: 10.2116/analsci.26.575.

(3) Cao, W.; Yang, J.-H.; Sun, C.-X.; Zhang, Z.-J.; Gao, Q.-F. Flow-injection–chemiluminescence method for the determination of penicillin G potassium. *Luminescence* **2005**, *20* (4-5), 238-242, https://doi.org/10.1002/bio.839. DOI: https://doi.org/10.1002/bio.839 (acccessed 2023/04/25).

(4) Sorouraddin, M. H.; Iranifam, M.; Naseri, A.; Fadakar-Sardroud, M.; Gharari-Alibabalou, H. Direct chemiluminescence determination of penicillin G potassium and a chemometrical optimization approach. *Luminescence* **2011**, *26* (6), 622-628, https://doi.org/10.1002/bio.1285. DOI: https://doi.org/10.1002/bio.1285 (acccessed 2023/04/25).

(5) Švorc, Ľ.; Sochr, J.; Rievaj, M.; Tomčík, P.; Bustin, D. Voltammetric determination of penicillin V in pharmaceutical formulations and human urine using a boron-doped diamond electrode. *Bioelectrochemistry* **2012**, *88*, 36-41. DOI: https://doi.org/10.1016/j.bioelechem.2012.04.004.
